# Mass production of two-dimensional oxides by rapid heating of hydrous chlorides

**DOI:** 10.1038/ncomms12543

**Published:** 2016-09-09

**Authors:** Chunsong Zhao, Haitian Zhang, Wenjie Si, Hui Wu

**Affiliations:** 1 State Key Laboratory of New Ceramics and Fine Processing, School of Materials Science and Engineering, Tsinghua University, Beijing 100084, China

## Abstract

Two-dimensional (2D) nanoscale oxides have attracted research interest owing to their electronic, magnetic optical and catalytic properties. If they could be manufactured on a large scale, 2D oxides would be attractive for applications ranging from electronics to energy conversion and storage. Herein, we report facile fabrication of oxide nanosheets by rapid thermal annealing of corresponding hydrous-chloride compounds. By heating CrCl_3_·6H_2_O, ZrOCl_2_·8H_2_O, AlCl_3_·6H_2_O and YCl_3_·6H_2_O crystals as precursors, we immediately collect large quantities of ultrathin Cr_2_O_3_, ZrO_2_, Al_2_O_3_ and Y_2_O_3_ nanosheets, respectively. The formation of layered nanosheets relies on exfoliation driven by rapid evaporation of water and/or other gas molecules generated under annealing. Our route allows simple, efficient and inexpensive production of 2D oxides. As a demonstration, we evaluate Cr_2_O_3_ nanosheets prepared by our method as anodes in lithium-ion batteries and find superior performance in comparison with their microcrystalline counterparts.

## Abstract (web summary)

Oxide nanosheets are of interest for a range of applications. Here the authors demonstrate a simple route combining gas evolution and exfoliation of hydrated chloride precursors for making large quantities of oxide nanosheets of various compositions.

## Graphical abstract

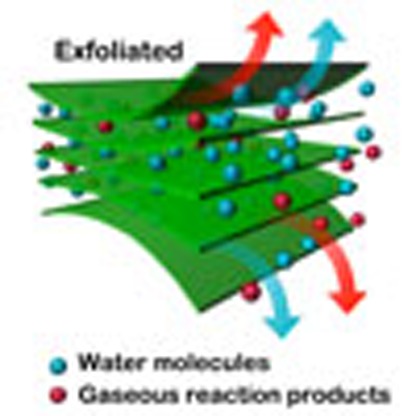

Materials of small geometrical dimensions have been successfully used in many technological applications because of their size-dependent physical and chemical properties^1^,^2^,^3^,^4^. In particular, the remarkable properties of graphene have renewed interest in inorganic two-dimensional (2D) materials with unique electronic, mechanical and optical attributes^5^,^6^,^7^,^8^,^9^,^10^. Beyond graphene, other 2D materials, such as transition metal dichalcogenides (for example, MoS_2_ and WS_2_), graphene analogues (for example, boron nitride), black phosphorus and some transition metal oxides (TMOs) (for example, MnO_2_ and TiO_2_) have attracted great attention because of their important applications in sensors, electrochemical catalysts and battery electrodes^11^,^12^,^13^,^14^,^15^,^16^,^17^,^18^.

Scientists have reported multiple methods of preparing 2D nanosheets. One of the most common strategies used to prepare 2D nanolayers is mechanical or chemical exfoliation; some layered materials, such as BN, MoS_2_, MoSe_2_, MoTe_2_ and TaSe, which have strong in-plane chemical bonds and weak out-of-plane van der Waals interactions, are easily exfoliated normal to the in-plane direction under extreme conditions^17^,^19^,^20^. A family of 2D nanosheets, labelled MXene, was produced through selective etching of the A-group atoms from MAX-phase solids such as Ti_3_AlC_2_ (refs 21, 22, 23, 24). Numerous 2D metal oxide nanosheets of materials such as TiO_2_, ZnO and MnO_2_ can be fabricated by self-assembly or other wet chemistry methods^16^,^25^. Some nanosheets without layered crystal structures can also be synthesized from starting materials such as metals. For example, researchers have prepared ultrathin metal nanosheets from materials such as rhodium and gold via solvothermal and wet chemistry methods, respectively^26^,^27^. Although researchers have studied many methods to synthesize 2D materials, these methods are usually complex, expensive and inefficient. The lack of chemical or physical approaches to synthesizing ultrathin nanosheets in large quantities has limited further development and applications of these 2D materials. Therefore, a simple, fast and economical method for the mass production of 2D nanomaterials remains an interesting challenge^28^.

In the liquid exfoliation method, ions or molecules are usually intercalated between layers to weaken the out-of-plane van der Waals interactions before the layer-structured materials are exfoliated^29^,^30^,^31^,^32^. Some layer-structured minerals with molecules between the layers also exist in nature, such as vermiculite. Vermiculite comprises a group of 2:1 phyllosilicate clay minerals that consist of hydrated sheet silicates that contain layers of water molecules within their internal structure^33^,^34^. When subjected to heat, vermiculite has the unusual property of expanding (commercial varieties can expand 8–20 times or more) because of the interlaminar generation of steam^33^,^34^. Accordingly, exfoliation of thin vermiculite layers occurs when the mineral is heated sufficiently (Supplementary Fig. 1).

Herein, inspired by the expansion and exfoliation that occur on heating of vermiculite, we apply rapid annealing to hydrous chlorides and oxychlorides and observe similar, large volume expansions. Using this approach, we successfully obtain sheets of layered oxides, including Cr_2_O_3_, ZrO_2_, Al_2_O_3_ and Y_2_O_3_. The thinnest of the exfoliated oxide layers was ∼1.2 nm thick (Supplementary Fig. 2) and spans several micrometres. Importantly, oxide nanosheets can be generated in large quantities by this rapid process. As a demonstration, Cr_2_O_3_ synthesized by our technique was evaluated as an anode material in the Li-ion batteries. The 2D structure provides a larger and more stable surface area than do microparticles, enhancing capacities accessible at useful rates. Moreover, Cr_2_O_3_ nanosheets show strong adhesion to the copper foil, even without binder, which enhances mechanical stability of electrodes during cycling.

## Results

### Materials design and synthesis

Some metal oxide solids cannot be synthesized by direct exfoliation methods because they do not have layered crystal structures. However, some chlorides or oxychlorides of the corresponding metal oxides possess layered structures. In addition, the chlorides and oxychlorides usually have water in the crystal lattice, such as CrCl_3_·6H_2_O, ZrOCl_2_·8H_2_O, AlCl_3_·6H_2_O and YCl_3_·6H_2_O. When the hydrous chlorides or oxychlorides are heated under dry conditions or/and at high temperatures, the following four chemical reactions occur:


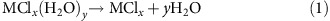



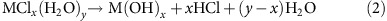



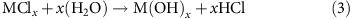



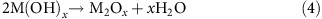


**Figure 1:**
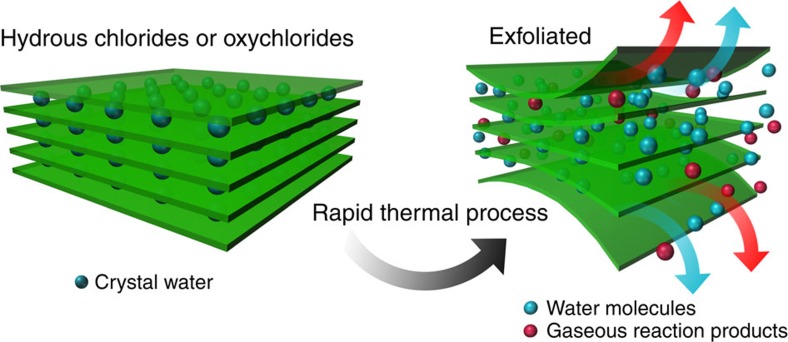
Material synthesis. Schematic of the exfoliation of hydrous chlorides. During heating, large quantities of gas (water or other gaseous reaction products) are released. The force generated by gas generation and expansion leads to the exfoliation of the hydrates.

During the dehydration and hydrolysis reactions in the rapid thermal process, a large quantity of gaseous reaction products, such as H_2_O and HCl, are produced within a short time, generating large pressures. The forces caused by gas evolution can drive exfoliation of layered solid products such as MCl_*x*_. The produced layers can further react spontaneously to form M_2_O_*x*_ nanosheets (Fig. 1a). Accordingly, we are able to obtain 2D metal oxide nanosheets from hydrated precursors via a rapid thermal process.

**Figure 2:**
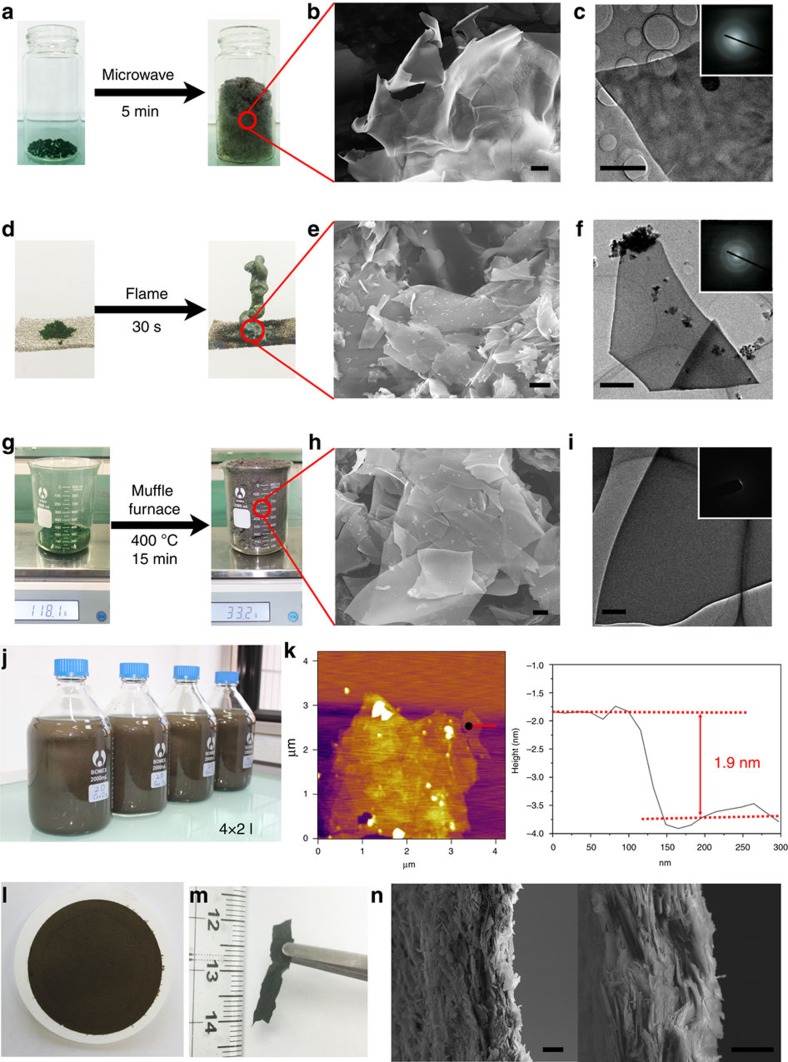
Nanosheets produced from chromium trichloride hexahydrate. (**a**) A CrCl_3_·6H_2_O sample before (left of panel) and after (right of panel) microwave heating. (**b**) SEM and (**c**) TEM images (inset, corresponding electron diffraction pattern) correspond to the sample in **a**. (**d**) A CrCl_3_·6H_2_O sample before (left of panel) and after (right of panel) flame heating. (**e**) SEM and (**f**) TEM images (inset, corresponding electron diffraction pattern) correspond to the sample in **d**. (**g**) A CrCl_3_·6H_2_O sample before (left of panel) and after (right of panel) being heated in the muffle furnace. (**h**) SEM and (**i**) TEM images (inset, corresponding electron diffraction pattern) correspond to the CrCl_3_·6H_2_O sample in **g**. (**j**) A large quantity (8 l) of the 2D Cr_2_O_3_ dispersion solution. (**k**) AFM characterization of the Cr_2_O_3_ obtained by alcohol lamp heating. (**l**) The 2D Cr_2_O_3_ film produced by vacuum filtration. (**m**) The free-standing 2D Cr_2_O_3_ film. (**n**) Cross-sectional SEM images of the free-standing 2D Cr_2_O_3_ film. Scale bars, 2 μm (**b**,**c**), 5 μm (**e**), 500 nm (**f**,**i**) and 10 μm (**h**,**n**).

Microwave heating is a common method used to heat or dry materials. The energy of the microwaves can be absorbed by water in a process called dielectric heating^35^, which is rapid and uniform. Accordingly, we used microwave heating to treat the CrCl_3_·6H_2_O precursor. The morphology of the CrCl_3_·6H_2_O particles is shown in a scanning electron microscopy (SEM) image (Supplementary Fig. 3); the particles were not layered nanosheets. The CrCl_3_·6H_2_O was placed in a glass bottle and then heated in a domestic microwave oven for 5 min. After heating, water was observed inside the bottle cap. The CrCl_3_·6H_2_O in the glass bottle underwent a very large volume expansion (Fig. 2a and Supplementary Movie 1). These observed phenomena were consistent with our expectations. The material obtained from CrCl_3_·6H_2_O through microwave heating is deliquescent and must be stored under dry conditions. We used SEM and transmission electron microscopy (TEM) to examine the sample (Fig. 2b,c). Typical 2D nanosheets with relatively large areas were observed. The diffraction pattern (Fig. 2c) indicated that the sample was amorphous. We used energy-dispersive spectroscopy (EDS) to detect the chemical elements in the sample. The resulting atomic ratio for Cr, O and Cl of 1:1:1 suggested that the sample was CrOCl (Supplementary Fig. 4a). The reason that we did not obtain Cr_2_O_3_ was likely insufficient heating power of the household microwave oven, leading to partial Cl removal. Although we collected CrOCl nanosheets using microwave heating, we investigated other rapid heating methods that could be used to obtain the oxides from CrCl_3_·6H_2_O.

The most immediate heating method is using a flame. The flame of an alcohol lamp is a dry environment and a high temperature (∼500 °C). We placed CrCl_3_·6H_2_O crystals on nickel foam and heated the crystals in the flame of an alcohol lamp for 30 s. We again observed marked volume expansion (Fig. 2d). Most of the obtained sample was insoluble in water. Typical 2D nanosheets with large area were observed by SEM (Fig. 2e) and TEM characterization (Fig. 2f). The nanosheets were amorphous according to the diffraction pattern (Fig. 2f) and the high-resolution TEM (HRTEM) image (Supplementary Fig. 5a). EDS analysis (Supplementary Fig. 5b) indicated that the nanosheets were mainly composed of Cr and O, but Cl was also present. On the basis of the EDS and X-ray photoelectron spectroscopy (XPS) results (Supplementary Fig. 5c), we posited that the obtained nanosheets were amorphous chromium oxide. However, it is difficult to distinguish between Cr_2_O_3_ and CrO_2_ using XPS results. Although we believe that a portion of the Cr in the nonstoichiometric amorphous product is high-valence Cr, for convenience, we refer to the materials as amorphous Cr_2_O_3_. The thickness of the Cr_2_O_3_ nanosheets was measured by atomic force microscopy (AFM). The thickness of the amorphous Cr_2_O_3_ obtained by alcohol lamp heating reached 1.9 nm (Fig. 2k). However, we also observed some sheets that were composed of small crystalline particles (Supplementary Fig. 5d,e). In addition, the X-ray diffraction pattern also suggested that the sample contained crystallographic Cr_2_O_3_ (Supplementary Fig. 5f). This result may be a consequence of the temperature distribution of the alcohol lamp flame being inhomogeneous, resulting in uneven heating of the CrCl_3_·6H_2_O crystals, which caused the sample to be non-uniform. We therefore changed the heating method from flame heating to a more homogeneous heating method to obtain uniform amorphous Cr_2_O_3_ nanosheets.

A muffle furnace can control temperature and maintain it at a constant level. This heating method is more homogeneous and capable of treating more samples. We set the temperature of the muffle furnace at 400 °C. The CrCl_3_·6H_2_O crystals were placed in a quartz crucible, directly placed into the furnace at 400 °C and treated for 15 min. After the heating process, the raw CrCl_3_·6H_2_O material exhibited large volume expansion (Fig. 2g and Supplementary Movie 2). Most of the obtained sample was insoluble in water, and large-area layered materials were observed by optical microscopy (Supplementary Fig. 6a). We observed a large quantity of 2D sheets by SEM (Fig. 2h). In addition, according to the diffraction pattern (Fig. 2i) and HRTEM results (Supplementary Fig. 6b), the nanosheets were amorphous. The EDS (Supplementary Fig. 6c) and XPS analysis results (Supplementary Fig. 6d) showed the samples mainly consisted of Cr and O with a small amount of Cl; the results also indicated that the obtained material was amorphous Cr_2_O_3_ nanosheets. Compared with the density of normal Cr_2_O_3_ (∼5.21 g ml^−1^), the tap density of the expanded Cr_2_O_3_ was very low, ∼0.0332, g ml^−1^. Using this method, we could mass-produce Cr_2_O_3_ nanosheets as shown in Supplementary Fig. 7a. For instance, we could fabricate 1,000 ml of Cr_2_O_3_ nanosheets in ∼2 h in the lab using only a small muffle furnace (the interior dimensions of the furnace are ∼10 × 10 × 10 cm^3^, as shown in Supplementary Fig. 7b). After removing the soluble impurities, the production yield of the Cr_2_O_3_ nanosheets is around 88%. We could also prepare 8 l of an aqueous Cr_2_O_3_ nanosheet solution at a concentration of 3 g l^−1^ (Fig. 2j). The Cr_2_O_3_ solution could be used to prepare free-standing films of Cr_2_O_3_ by vacuum filtration (Fig. 2l,m). Figure 2n shows a cross-section of the free-standing Cr_2_O_3_ films. Such solution-based methods are favourable for practical applications such as preparing and processing electrode slurries. In addition to the rapid heating treatment, we also annealed CrCl_3_·6H_2_O crystals under a gradually increasing temperature profile; samples were heated from room temperature to 400 °C at 5 °C min^−1^ in a muffle furnace and maintained at 400 °C for 15 min. We observed the expected volume expansion, but we did not observe predominant quantities of smooth, large-area nanosheets. Most of the resulting sample was in the form of thick sheets formed by small particles (Supplementary Fig. 8). This result suggests that the hydrous chloride crystals could be exfoliated and further reacted to form oxides by heat treatment. However, layer thickness may be influenced by the rate of temperature increase; the exfoliated oxide sheets gradually crystallize to form small Cr_2_O_3_ crystal particles during the slow heat-treatment process. Accordingly, the rate of temperature increase during the heating treatment is key to obtaining ultrathin 2D metal oxide nanosheets.

### Electrochemical performance of the Cr_2_O_3_ nanosheets

To explore the application of our exfoliated 2D Cr_2_O_3_ nanosheets, we incorporated the amorphous Cr_2_O_3_ nanosheets into Li-ion batteries. Lithium can be stored reversibly in TMOs through the following reaction:


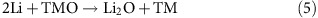


Among TMOs, Cr_2_O_3_ is suitable as an anode material for Li-ion batteries because of its high theoretical capacity (1,058 mAh g^−1^), low average charging voltage (∼1.2 V) and low electromotive force value (1.085 V versus Li/Li^+^)^36^,^37^. However, the cycling performance of Cr_2_O_3_ electrodes is poor because of a loss of electronic contact of active materials and poor electron and ion transport properties within the active material^36^. We investigated whether use of our 2D Cr_2_O_3_ nanosheets as active materials could address these limitations. We fabricated graphene/2D Cr_2_O_3_ composite electrodes. The graphene and Cr_2_O_3_ were assembled layer by layer because of their similar 2D layered structure. The stacked 2D structure can provide good electrical contact between the graphene and Cr_2_O_3_ nanosheets, improving electron transport. The ultrathin, 2D layered structure can help access to the active material by the electrolyte and aid ion transport.

**Figure 3:**
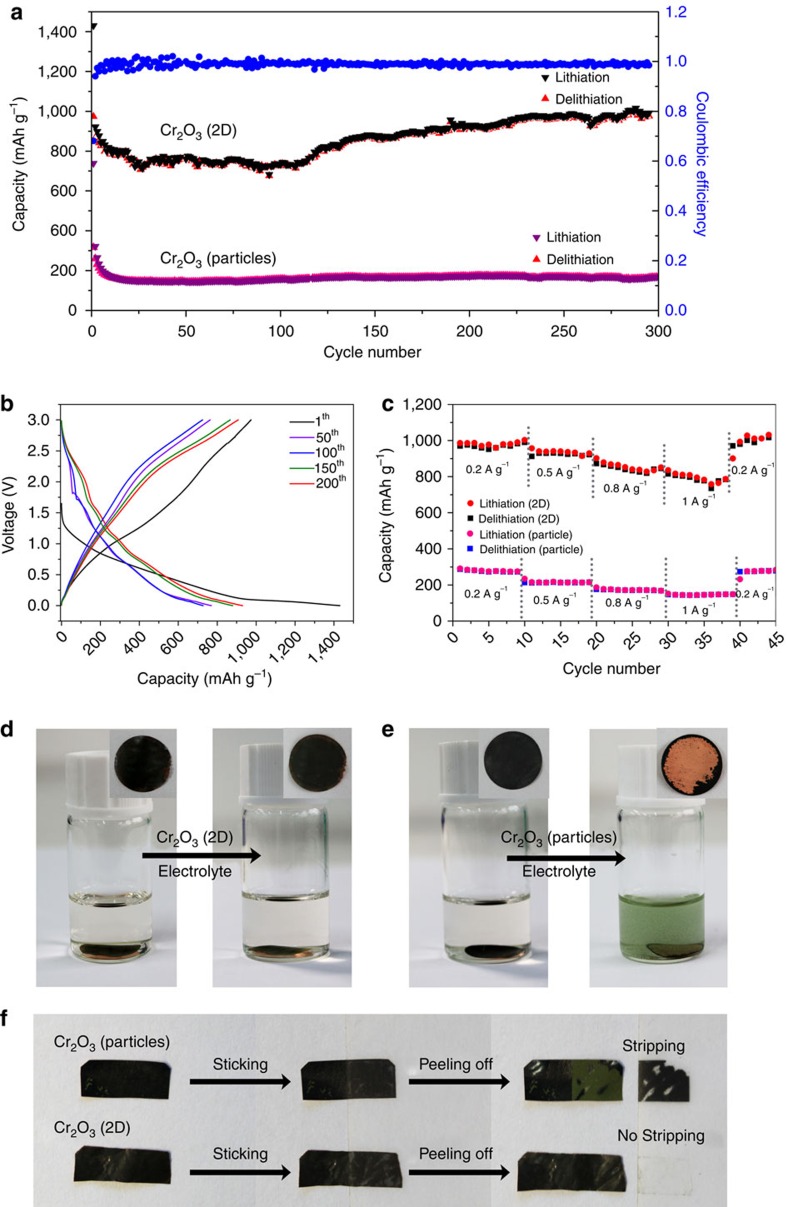
Electrochemical and adhesion performance of chromic oxides. (**a**) The cycling performance of the Cr_2_O_3_ electrodes. (**b**) The galvanostatic lithiation/delithiation profiles of the 2D Cr_2_O_3_ electrodes. (**c**) The rate performance of the Cr_2_O_3_ electrodes. (**d**) The Cr_2_O_3_ (2D) electrode and (**e**) Cr_2_O_3_ (particles) electrode before (left of panel) and after (right of panel) being ultrasonically cleaned in an electrolyte solution. (**f**) The Cr_2_O_3_ electrodes before and after mechanical exfoliation.

We fabricated graphene/Cr_2_O_3_ (2D) electrodes and graphene/Cr_2_O_3_ (particle)/polyvinylidene difluoride (PVDF) electrodes, as mentioned in the Methods section. The cells were tested over a voltage range from 0 to 3 V versus Li^+^/Li. During the test, the initial lithiation and delithiation capacity of the graphene/Cr_2_O_3_ (particle)/PVDF electrodes was only ∼738 and 316 mAh g^−1^, respectively, and the delithiation capacity decreased to only 168 mAh g^−1^ after ∼10 cycles at the current density of 0.2 A g^−1^ (Fig. 3a). By contrast, the graphene/Cr_2_O_3_ (2D) electrodes exhibited a high capacity and stable cycling performance. The graphene/Cr_2_O_3_ (2D) electrodes achieved an initial delithiation capacity of 974 mAh g^−1^ at a rate of 0.2 A g^−1^. After 297 cycles, the capacity was ∼986 mAh g^−1^, and the cell continued to function well (Fig. 3a). This electrochemical performance of the graphene/Cr_2_O_3_ (2D) electrodes is excellent compared with the reported performance of other Cr_2_O_3_ electrodes^38^,^39^,^40^,^41^,^42^. The galvanostatic lithiation/delithiation profiles and rate performance of our 2D Cr_2_O_3_ electrodes are included in Fig. 3b,c. We measured the impedance of the electrodes by electrochemical impedance spectroscopy in terms of Nyquist plots (Supplementary Fig. 9). The semicircle in high-frequency region is related to the solid electrolyte interphase (SEI) film and the medium-frequency semicircle due to the charge transfer resistance, and the inclined line in the low-frequency region represents the diffusion of lithium ions^43^,^44^. The surface film resistance, which originates from the SEI, and the charge transfer resistance of the graphene/Cr_2_O_3_ (particle)/PVDF electrode were 12.3 and 56.9 Ω, respectively. However, the graphene/Cr_2_O_3_ (2D) composites showed lower SEI and charge transfer resistances, which were 6.6 and 21.4 Ω. The electrochemical impedance spectroscopy results indicated the interface of graphene/Cr_2_O_3_ (2D) electrodes is more stable and confirmed the 2D structure is attributed to the charge transfer and charge diffusion, which could support the good electrochemical and rate performances of graphene/Cr_2_O_3_ (2D) electrodes. Importantly, the graphene/Cr_2_O_3_ (2D) composites exhibited strong adhesion to the current collector (copper foil) and between the layers. The graphene/Cr_2_O_3_ (2D) electrodes and graphene/Cr_2_O_3_ (particle)/PVDF electrodes were immersed in the electrolyte solution; we then sonicated the electrodes in the electrolyte solution with ultrasonic cleaning (80 W, 40 kHz) for 5 min. For the graphene/Cr_2_O_3_ (particle)/PVDF electrodes, the PVDF binder did not prevent the spalling of the electrode material from the current collector during the ultrasonic cleaning process (Fig. 3e). However, the graphene/Cr_2_O_3_ (2D) composite materials remained on the current collector (Fig. 3d). We also tested the adhesion of the two electrode types by cleaning them ultrasonically in water and ethyl alcohol. The graphene/Cr_2_O_3_ (2D) electrodes maintained structural stability in all three solutions, whereas the graphene/Cr_2_O_3_ (particle)/PVDF electrodes exhibited different degrees of spalling (Supplementary Fig. 10). We used the scotch tape method to mechanically remove material from the electrodes. While the graphene/Cr_2_O_3_ (particle)/PVDF electrode was easily peeled off, the graphene/Cr_2_O_3_ (2D) electrodes remained intact and no stripping happened (Fig. 3f and Supplementary Movie 3). The observed strong adhesion and the structural stability of our graphene/2D Cr_2_O_3_ composite materials could explain the stable electrochemical performance of the graphene/Cr_2_O_3_ (2D) electrodes. We suggest the strong adhesion has three potential causes. First, the 2D structure of Cr_2_O_3_ provides a high contact area with graphene and copper foil, and the calendaring process ensures good contact between Cr_2_O_3_ (2D), graphene and copper foil. Second, according to zeta potential measurements under neutral conditions, graphene was negatively charged (−9.26 mV) and the 2D Cr_2_O_3_ was positively charged (32.5 mV), suggesting that electrostatic attraction contributes to the adhesion between 2D Cr_2_O_3_ and graphene. Third, and finally, we speculate that some chemical bonding may occur at the interface between the electrode material and current collector, between amorphous Cr_2_O_3_ nanosheets and the copper foil due to the high-valence Cr and low-coordinated surface atoms, leading to robust adhesion to the copper foil^45^,^46^. Such adhesion properties are interesting for applications in many fields, such as flexible energy storage devices or chemical coatings.

### Extended material systems

**Figure 4:**
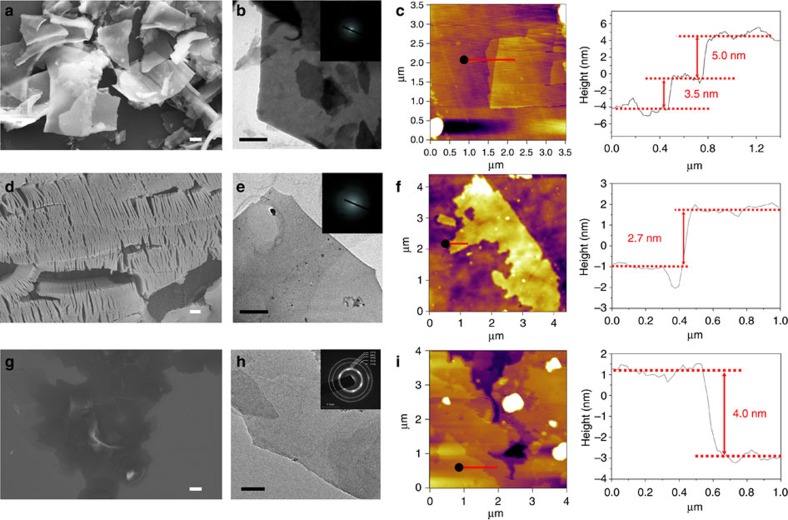
Characterizations of the oxide nanosheets. (**a**) SEM, (**b**) TEM (inset, corresponding electron diffraction pattern) and (**c**) AFM characterizations of the ZrO_2_ nanosheets. (**d**) SEM, (**e**) TEM (inset, corresponding electron diffraction pattern) and (**f**) AFM characterizations of the Al_2_O_3_ nanosheets. (**g**) SEM, (**h**) TEM (inset, corresponding electron diffraction pattern) and (**i**) AFM characterizations of the Y_2_O_3_ nanosheets. Scale bars, 2 μm (**a**,**d**,**g**), 500 nm (**b**,**e**) and 50 nm (**h**).

Beyond the chromium oxides from the CrCl_3_·6H_2_O, we applied the rapid heating method to other hydrous chlorides to obtain metal oxides such as ZrO_2_, Al_2_O_3_ and Y_2_O_3_. The ZrOCl_2_·8H_2_O, AlCl_3_·6H_2_O and YCl_3_·6H_2_O crystals were not layered nanosheets before the rapid heating treatment (Supplementary Fig. 11). The ZrOCl_2_·8H_2_O starting material was heated in the microwave oven for 10 min. A portion of the obtained sample dissolved in water, and we collected the insoluble portion. Typical nanosheets were obtained, as shown by optical microscopy (Supplementary Fig. 12a), SEM (Fig. 4a) and TEM images (Fig. 4b). On the basis of the EDS (Supplementary Fig. 12c) and XPS (Supplementary Fig. 12d) results, we deduced the nanosheets to be ZrO_2_. The diffraction pattern (Fig. 4b) and HRTEM results (Supplementary Fig. 12b) indicated that the 2D ZrO_2_ sheets were amorphous.

In the case of the AlCl_3_·6H_2_O crystals, we placed the crystals in a glass bottle and heated them for ∼5 min using the alcohol lamp. After the heat treatment, the exfoliated sample was observed by SEM (Supplementary Fig. 13), although inidividual sheets were difficult to distinguish. We therefore chose an alternative method to produce Al_2_O_3_ sheets. We used a heating gun to generate hot air with a maximum temperature of ∼500 °C. We prepared an AlCl_3_ solution (1 g ml^−1^) in water and painted the solution onto copper foil. The copper foil was then dried in an oven at 60 °C for ∼30 min to obtain a hydrous AlCl_3_ thin film. We heated the hydrous AlCl_3_ on the copper foil using the heating gun (∼500 °C) until the light-yellow solid appeared. We observed that the solid formed on the copper foil was exfoliated Al_2_O_3_ (Fig. 4d); ultrathin nanosheets were observed using TEM (Fig. 4e). These sheets were amorphous Al_2_O_3_ according to the diffraction pattern (Fig. 4e), HRTEM, EDS and XPS results (Supplementary Fig. 14).

We also produced Y_2_O_3_ nanosheets using the rapid heat-treatment furnace, which could heat to hundreds of degrees within a few seconds. The heating principle of the rapid heat treatment furnace is light radiation, and the process generates strong air flow. The light radiation heats the hydrous chlorides and causes water and hydrogen chloride molecules to escape from the crystals. The air flow removes the water and hydrogen chloride molecules from the environment, which favours the escape of more gas molecules. We treated YCl_3_·6H_2_O samples in this furnace. The YCl_3_·6H_2_O was placed in a crucible, heated to 600 °C, and maintained at this temperature for 2 min in the rapid heat-treatment furnace. Although the samples were not uniform and contained numerous particles, we still obtained Y_2_O_3_ nanosheets, as indicated by SEM, TEM (Fig. 4g,h) and EDS results (Supplementary Fig. 15b). The diffraction pattern (Fig. 4h) and HRTEM (Supplementary Fig. 15a) results indicated that part of the Y_2_O_3_ nanosheets was crystallized (PDF#44–0399), but the crystallinity was poor. We measured the thickness of the metal oxide nanosheets using AFM. The thinnest layers of the ZrO_2_, Al_2_O_3_ and Y_2_O_3_ obtained by rapid heating methods exhibited layer thicknesses of 3.5, 2.7 and 4.0 nm, respectively (Fig. 4c,f,i).

## Discussion

We could collect the 2D nanosheets using different combinations of the aforementioned materials and heating methods. For example, we obtained amorphous Cr_2_O_3_ nanosheets by heating the CrCl_3_·6H_2_O in the rapid heat treatment furnace (Supplementary Fig. 16). Some precursors without crystal water (such as anhydrous AlCl_3_) or without layered crystal structure (such as AlNO_3_·9H_2_O) were also treated by the rapid heating process, however the 2D oxides were not obtained in these cases (Supplementary Figs 17 and 18), which strengthens our point about the formation mechanism of oxide nanosheets. Although we obtained metal oxides using the various methods previously mentioned, the idea of treating the hydrous chloride with a rapid thermal process is consistent. The differences are the heating principles and specific procedures. However, the influential factors and manufacturing techniques require further improvement and study because the products obtained were not uniform and because we could not produce other TMOs to the same degree as we could produce the Cr_2_O_3_ nanosheets.

We produced the 2D Cr_2_O_3_ nanosheets on a large scale within a short time (that is, several minutes) by rapidly heating hydrous chlorides; the 2D Cr_2_O_3_ showed excellent electrochemical performance in Li-ion battery and surprisingly strong adhesion to the copper foil substrate. We also demonstrated that this method of producing ultrathin nanosheets could be generalized to allow rapid production of other oxides such as ZrO_2_, Al_2_O_3_ and Y_2_O_3_. We believe that this concept provides a practically promising avenue for simple, efficient, fast and inexpensive production of large quantities of large-area, ultrathin 2D nanosheets.

## Methods

### Synthesis of 2D CrOCl by microwave heating

The CrCl_3_·6H_2_O crystals were placed in a glass bottle and heated using a microwave oven for ∼5 min. Then, the obtained CrOCl materials were transferred and stored in an Ar-filled glove box. The CrOCl nanosheets were dispersed in benzene to prepare TEM samples.

### Synthesis of the 2D oxides

The CrCl_3_·6H_2_O crystals were placed on nickel foam and heated using an alcohol lamp for ∼2 min; or placed in a quartz crucible and heated in a muffle furnace at 400 °C for ∼15 min. The ZrOCl_2_·8H_2_O crystals were placed in a glass bottle and heated using a microwave oven for ∼10 min. The AlCl_3_·6H_2_O crystals were dissolved in deionized water at a concentration of 1 g ml^−1^, and the solution was painted onto copper foil. The copper foil was dried in an oven at 60 °C for 30 min to obtain the hydrous AlCl_3_ thin film. We heated the hydrous AlCl_3_ on the copper foil using a heating gun until a white or light-yellow solid appeared. The white or light-yellow solid was Al_2_O_3_ nanosheets. The YCl_3_·6H_2_O crystals were placed in an alumina crucible and heated in the rapid heat treatment furnace at 600 °C for ∼2 min. The obtained metal oxide products contain incompletely reacted intermediate products. We dispersed the obtained products in deionized water to remove the soluble impurities. The dispersion of products was centrifuged for 5 min at 12,000 r.p.m. to obtain the sediment. We repeated the centrifuging process three times to clean the products (the first two times using deionized water and the third time using ethyl alcohol). The obtained sediment was dried in an oven at 80 °C.

### Battery electrodes based on nanosheets or on microparticles

We dispersed graphene (10 wt%) and Cr_2_O_3_ nanosheets (90 wt%) in water at a concentration of 10 mg ml^−1^ (total mass). We also dispersed graphene (10 wt%), Cr_2_O_3_ particles (∼300 nm, 80 wt%) and PVDF (10 wt%) in N-methyl-2-pyrrolidone (NMP) at a concentration of 10 mg ml^−1^ (total mass). The composite slurry was dropped onto a flat copper foil and dried in an oven at ∼80 °C. The dried electrodes were pressed using a calendaring process (∼20 MPa). The mass loading of the electrodes was ∼0.5 mg cm^−2^. The electrodes were assembled into half-cells with Li metal foil (MTI) as a counter electrode in an Ar-filled glove box. We used a 25 μm-thick microporous polypropylene membrane as the separator (Asahi Kasei) and 1 M LiPF_6_ in ethylene carbonate/diethyl carbonate/fluoroethylene carbonate (1:1:0.04 vol/vol/vol, Ferro Corporation) as the electrolyte.

### Characterization

The X-ray diffraction patterns of the rare materials and products were evaluated using a D/max-2500 diffractometer (Rigaku, Japan) equipped with a CuK_α_ radiation source. The chemical states of the products were determined by XPS (Thermo Fisher ESCALAB 250Xi). The morphology of the samples was observed with a SEM (MERLIN VP Compact, ZEISS, Germany). The chemical composition of the samples was analysed using EDS (X-Max^N^, Oxford Instruments). HRTEM observation was carried out using JEOL-2100 TEM operated at 200 kV. The thickness of the nanosheets was determined by AFM (MFP-3D, Asylum Research, Oxford Instruments). The electrochemical performances of the batteries were measured by a BS-9300R/10V2A MTI 8 channels battery analyser.

### Data availability

The data that support the findings of this study are available from the corresponding author on request.

## Additional information

**How to cite this article:** Zhao, C. *et al*. Mass production of two-dimensional oxides by rapid heating of hydrous chlorides. *Nat. Commun.* 7:12543 doi: 10.1038/ncomms12543 (2016).

## Supplementary Material

Supplementary Information Supplementary Figures 1-18

Supplementary Movie 1 CrCl_3_˙6H_2_O starting material undergoes large volume expansion upon microwave heating.

Supplementary Movie 2 CrCl_3_˙6H_2_O crystals directly placed into a furnace at 400 C and heated for 5 minutes to obtain porous 2D Cr_2_O_3_ nanomaterials.

Supplementary Movie 3 Tape test showing strong adhesion between Cr_2_O_3_ nanosheets and copper foil substrate.
