# Supplementary material for: Mass production of two-dimensional oxides by rapid heating of hydrous chlorides
Source: Nat Commun. 2016 Sep 9;7:12543. doi: 10.1038/ncomms12543 (PMC5023967; doi:10.1038/ncomms12543)
Supplement: Supplementary Information — Supplementary Figures 1-18 [file ncomms12543-s1.pdf]

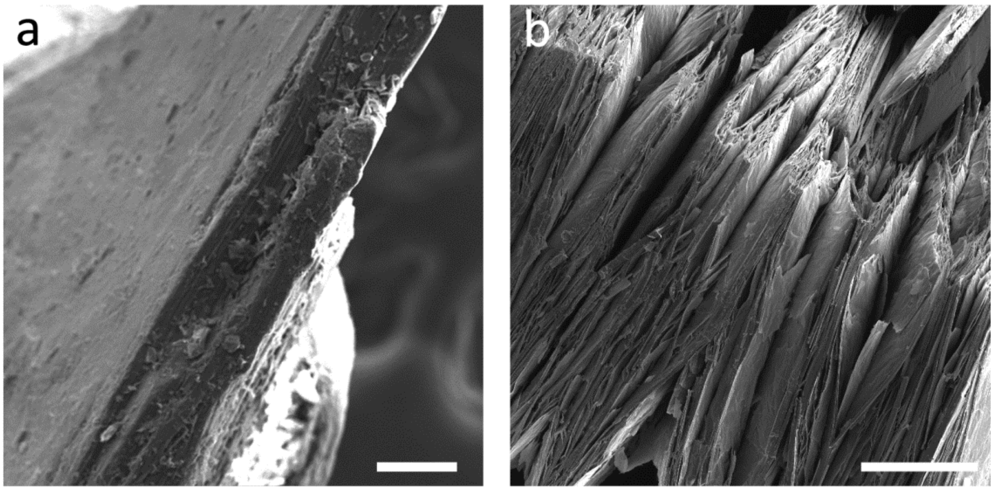

Supplementary Figure 1 | SEM of the vermiculite samples. Vermiculite (**a**) before and (**b**) after heat treatment. Scale bars, 100 μm (**a**) and 500 μm (**b**).

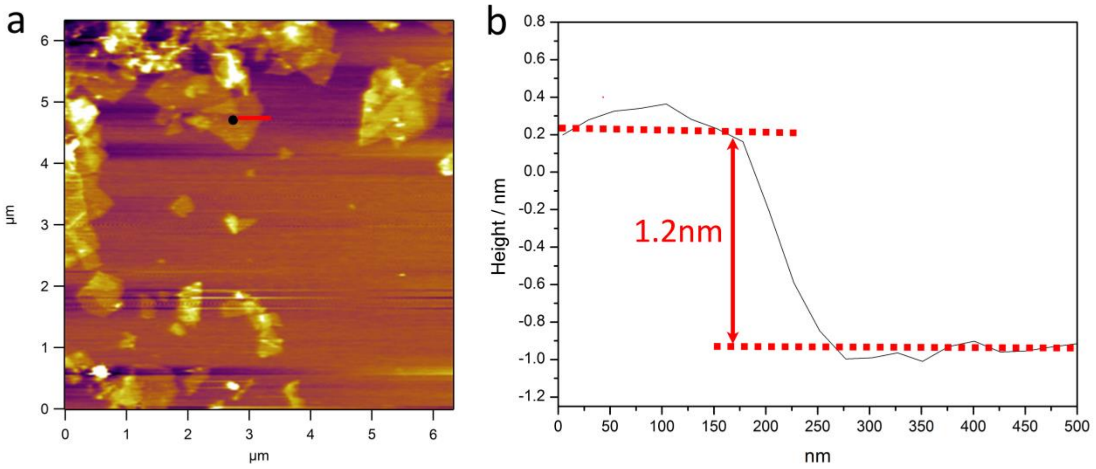

Supplementary Figure 2 | Thickness of Cr_2O_3 nanosheet measured by AFM. AFM image of the Cr_2O_3 obtained by alcohol lamp heating (**a**), (**b**) Height profile along the red line indicated in (**a**), which is consistent with a sheet thickness of 1.2 nm.

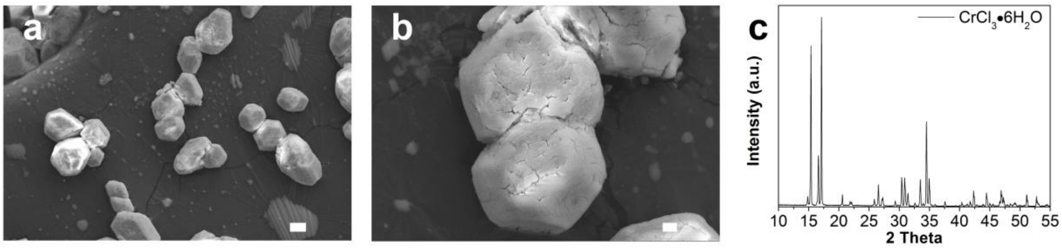

Supplementary Figure 3 | Characterization of $CrCl_3{\cdot}6H_2O$ starting material. (**a,b**) SEM images and (**c**) XRD pattern of the $CrCl_3{\cdot}6H_2O$ raw material. Scale bar, 10 μm (**a**) and 2 μm (**b**).

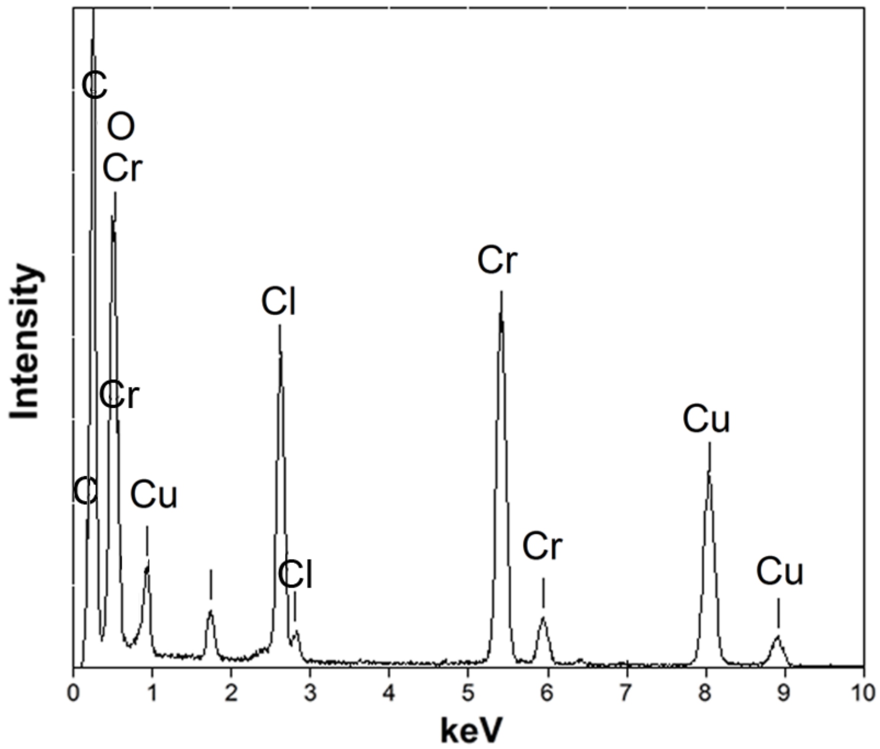

Supplementary Figure 4 | EDS of material produced by microwave heating. EDS analysis results suggested the sample is CrOCl (obtained by heating $CrCl_3 \cdot 6H_2O$ in a microwave oven).

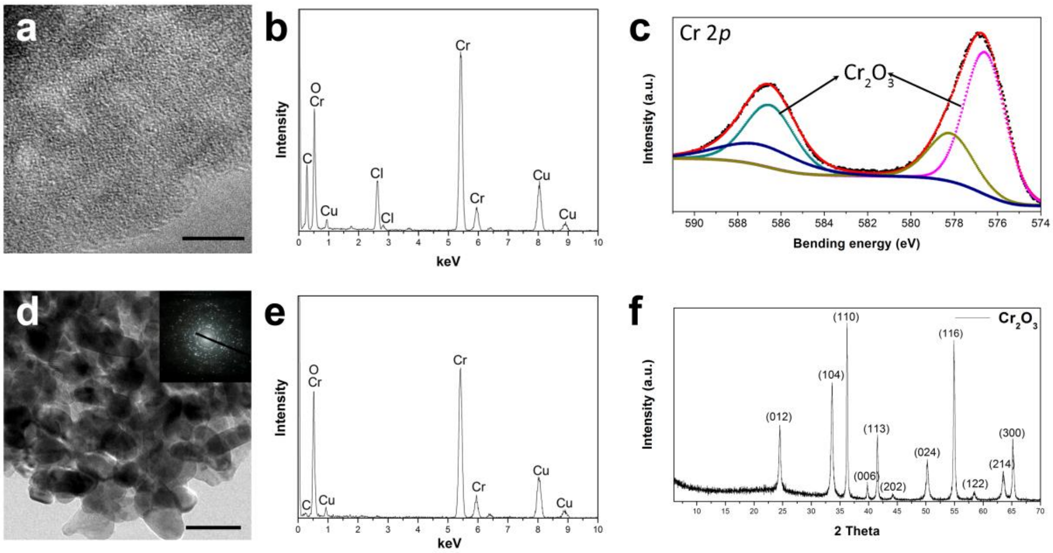

Supplementary Figure 5 | Characterization of Cr_2O_3. The Cr_2O_3 was obtained by heating $CrCl_3{\cdot}6H_2O$ using an alcohol lamp. (**a**) HRTEM image of the Cr_2O_3 nanosheets. (**b**) EDS result for the samples shown in (**a**). (**c**) XPS results of the Cr_2O_3. (**d**) TEM image of a sheet formed by Cr_2O_3 crystal particles (Inset) Corresponding electron diffraction pattern. (**e**) EDS result for the Cr_2O_3 shown in (**d**). (**f**) XRD pattern of the Cr_2O_3 sample. Scale bar, 5 nm (**a**) and 100 nm (**d**).

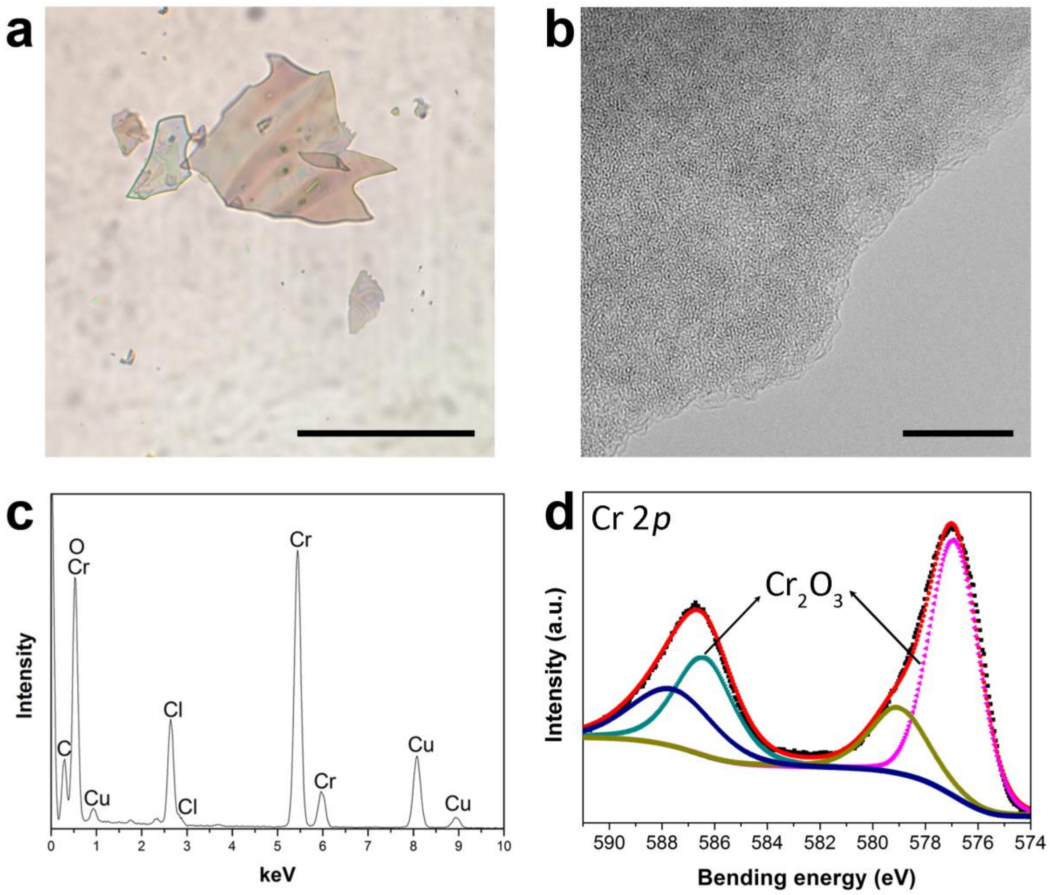

Supplementary Figure 6 | Characterization of Cr_2O_3. The Cr_2O_3 was obtained by heating $CrCl_3 \cdot 6H_2O$ using the muffle furnace**.** (**a**) Optical microscopy image of Cr_2O_3. (**b**) HRTEM image of Cr_2O_3 nanosheets. (**c**) EDS result for the Cr_2O_3. (**d**) XPS result of the Cr_2O_3. Scale bar, 50 μm (**a**) and 10 nm (**b**).

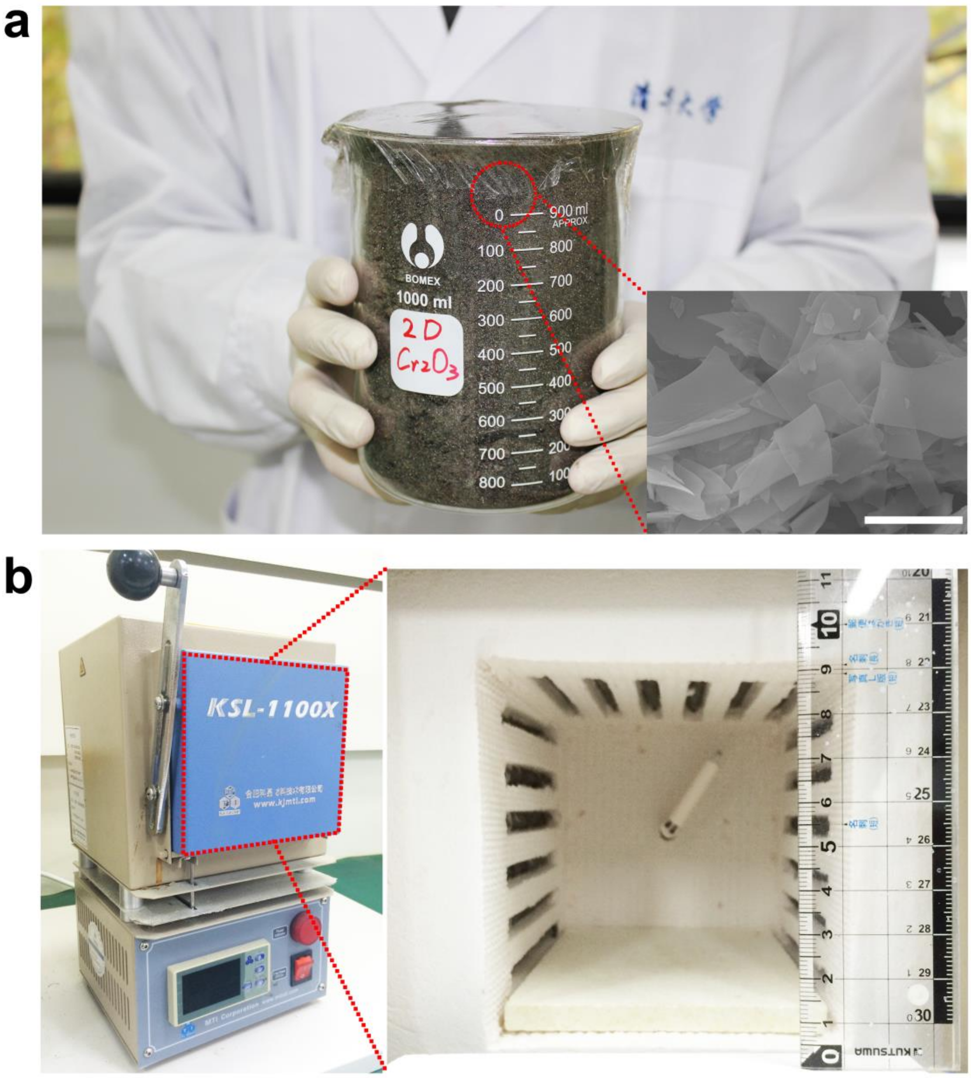

Supplementary Figure 7 | Large-scale production. (**a**) 1000 ml batch of 2D chrome oxide nanosheets. (Inset) SEM image of the chromium oxide nanosheets. Scale bar, 10 μm. (**b**) The muffle furnace used in the experiments. The interior dimensions of the furnace are approximately 10 × 10 × 10 cm^3.

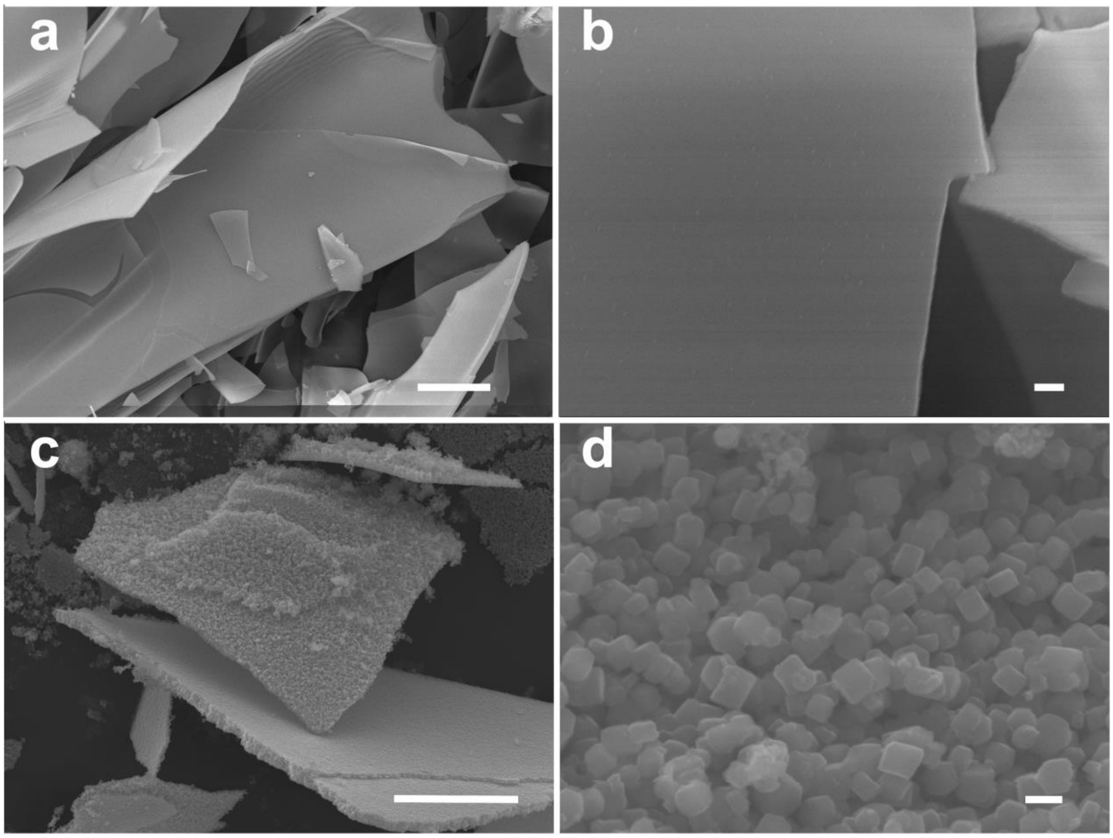

Supplementary Figure 8 | SEM images of Cr_2O_3. (**a,b**) The Cr_2O_3 obtained by heating $CrCl_3 \cdot 6H_2O$ in a muffle furnace at 400 °C for 15 minutes without a temperature-ramping period. (**c,d**) The Cr_2O_3 obtained by heating $CrCl_3 \cdot 6H_2O$ in a muffle furnace at 400 °C for 15 minutes with a temperature ramp rate of 5 °C/min. Scale bar, 10 μm (**a,c**) and 200 nm (**b,d**).

a

b

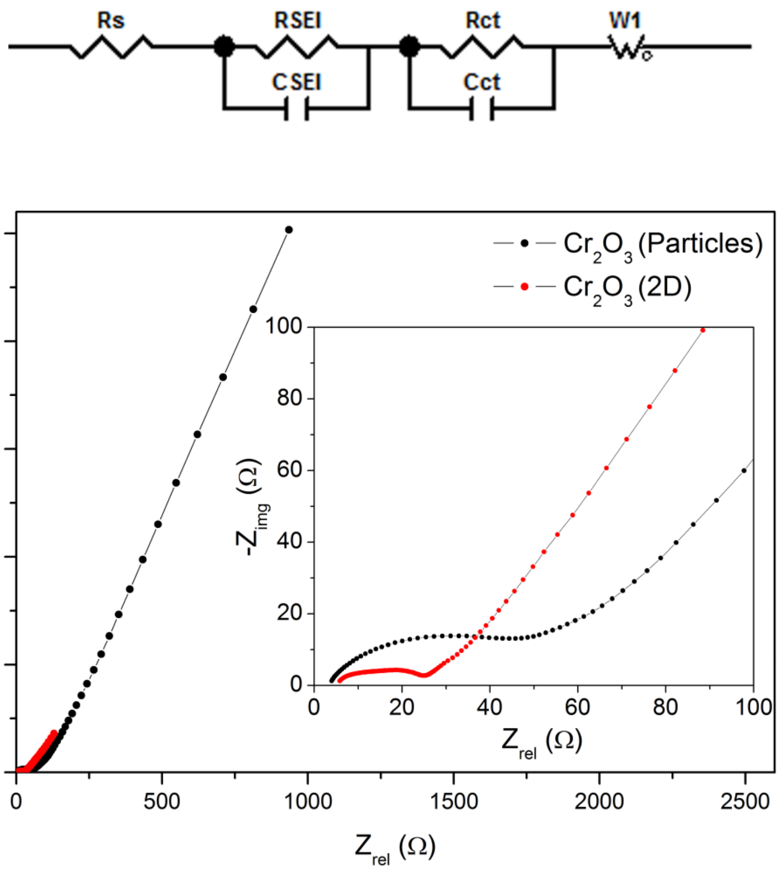

Supplementary Figure 9 | The impedance of the electrodes. (**a**) Equivalent circuit used for fitting the impedance spectra of the electrodes. (**b**) Nyquist plots of EIS spectra of the graphene/Cr_2O_3 (2D) electrodes and graphene/ Cr_2O_3 (particle)/PVDF electrodes in delithiation stage.

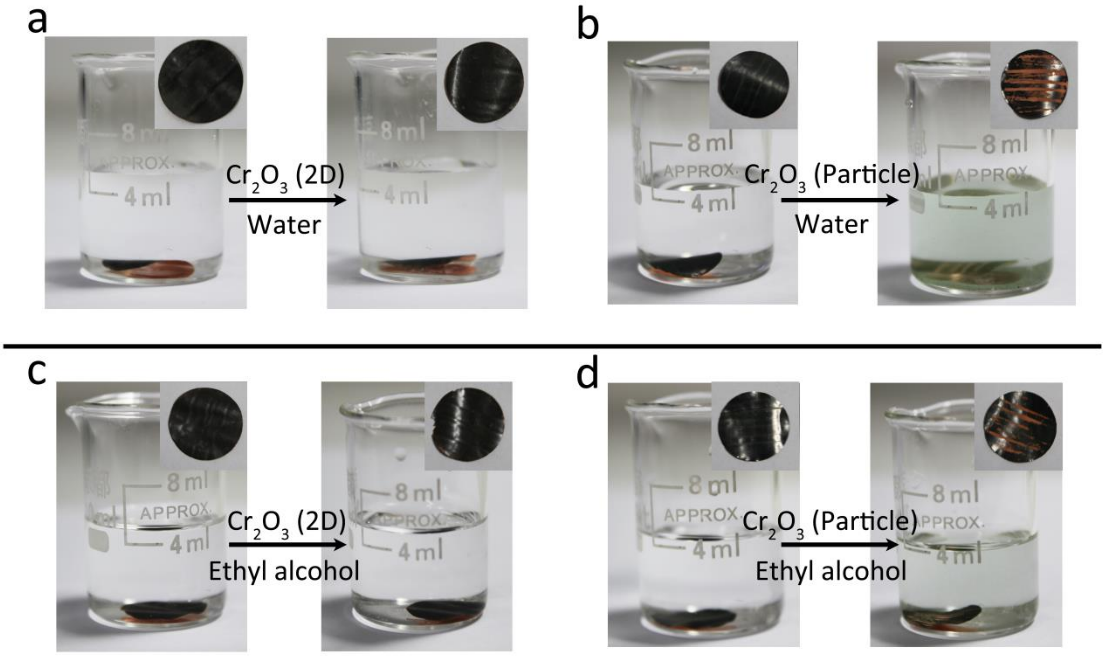

Supplementary Figure 10 | Adhesion performance of Cr_2O_3 (2D and particles). The 2D Cr_2O_3/graphene layers showed strong adhesion to the current collector (copper foil) as well as between layers. Panels (a) and (b) show the (**a**) Cr_2O_3 (2D)/graphene electrode and (**b**) Cr_2O_3 (particle)/graphene/PVDF electrode before (left of panel) and after (right of panel) they were ultrasonically cleaned in water for 5 minutes. Panels (**c**) and (**d**) show the Cr_2O_3 (2D)/graphene electrode and Cr_2O_3 (particle)/graphene/PVDF electrode before (left of panel) and after (right of panel) they were ultrasonically cleaned in ethyl alcohol for five minutes. The Cr_2O_3 (2D)/graphene electrode remained intact after being ultrasonically cleaned in water or ethyl alcohol. However, the Cr_2O_3 (particle)/graphene/PVDF electrode separated from the copper foil after ultrasonication.

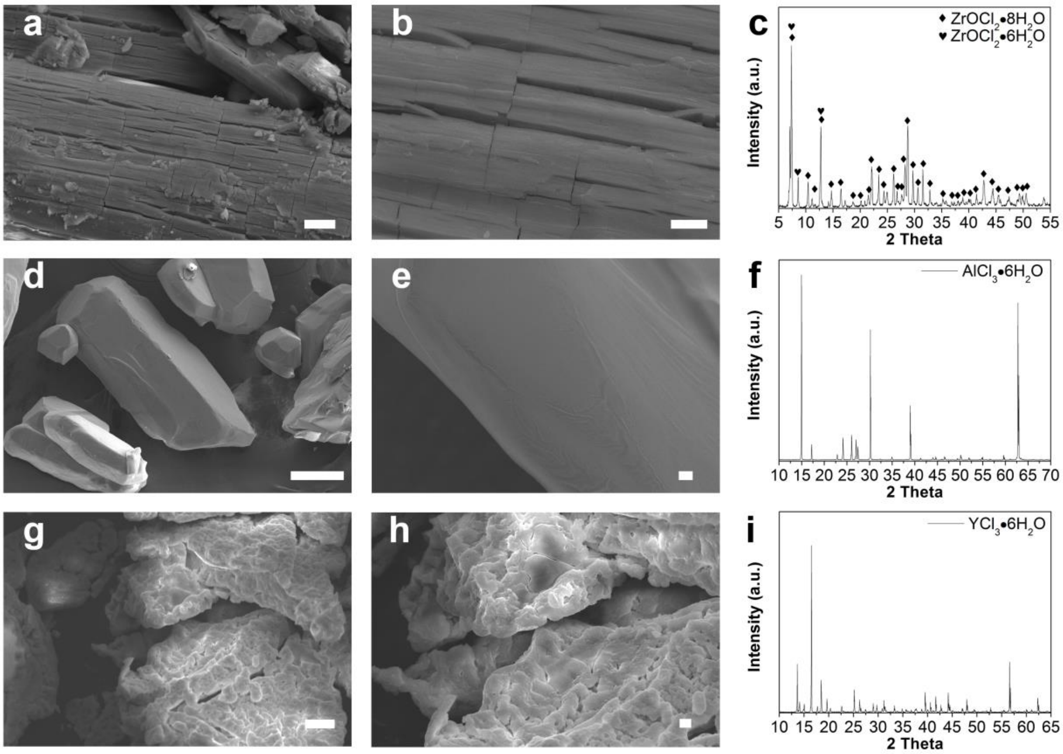

Supplementary Figure 11 | Characterization of raw materials. (**a,b**) SEM images and (**c**) XRD pattern of the $ZrOCl_2 \cdot 8H_2O$ raw material including few $ZrOCl_2 \cdot 6H_2O$. (**d,e**) SEM images and (**f**) XRD pattern of the $AlCl_3 \cdot 6H_2O$ raw material. (**g,h**) SEM images and (**i**) XRD pattern of the $YCl_3 \cdot 6H_2O$ raw material. Scale bar 10 μm (**a,g**), 2 μm (**b,e,h**) and 100 μm (**d**).

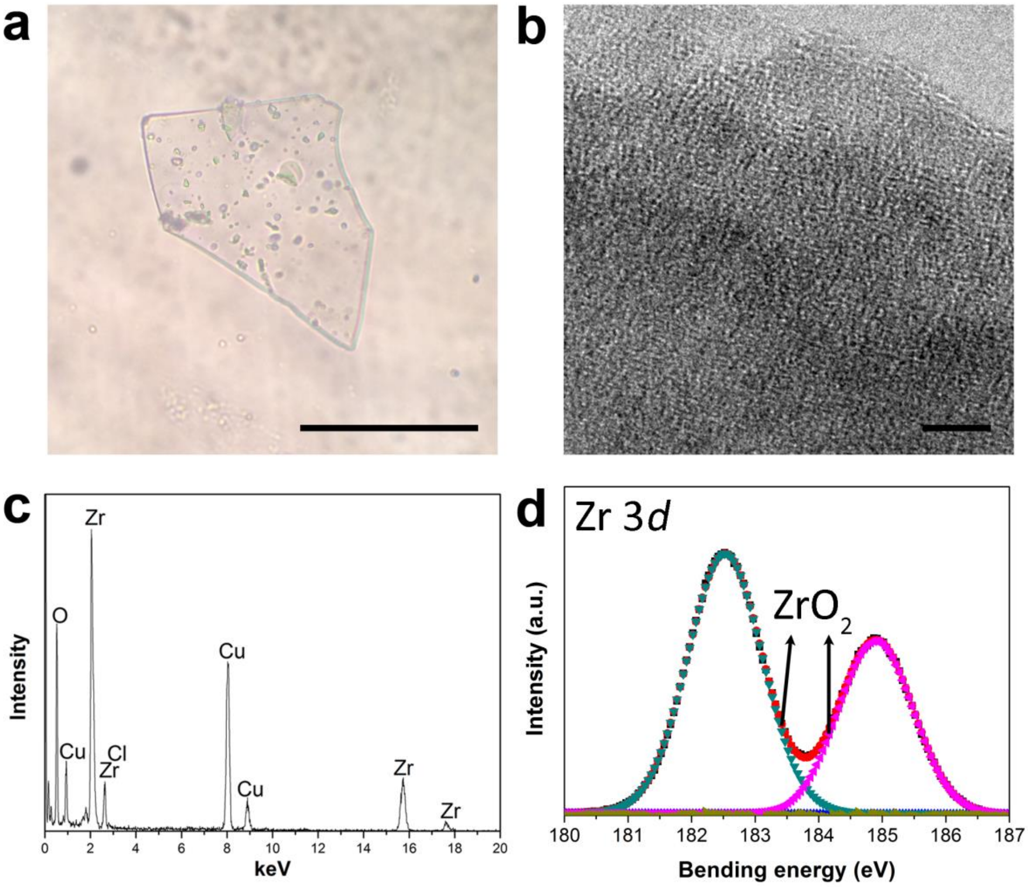

Supplementary Figure 12 | Characterization of ZrO_2. ZrO_2 was obtained by heating $ZrOCl_2 \cdot 8H_2O$ using a microwave oven**.** (**a**) Optical microscopy images of ZrO_2. (**b**) HRTEM image of ZrO_2 nanosheets. (**c**) EDS result for the ZrO_2. (**d**) XPS result of the ZrO_2. Scale bar, 50 μm (**a**) and 5 nm (**b**).

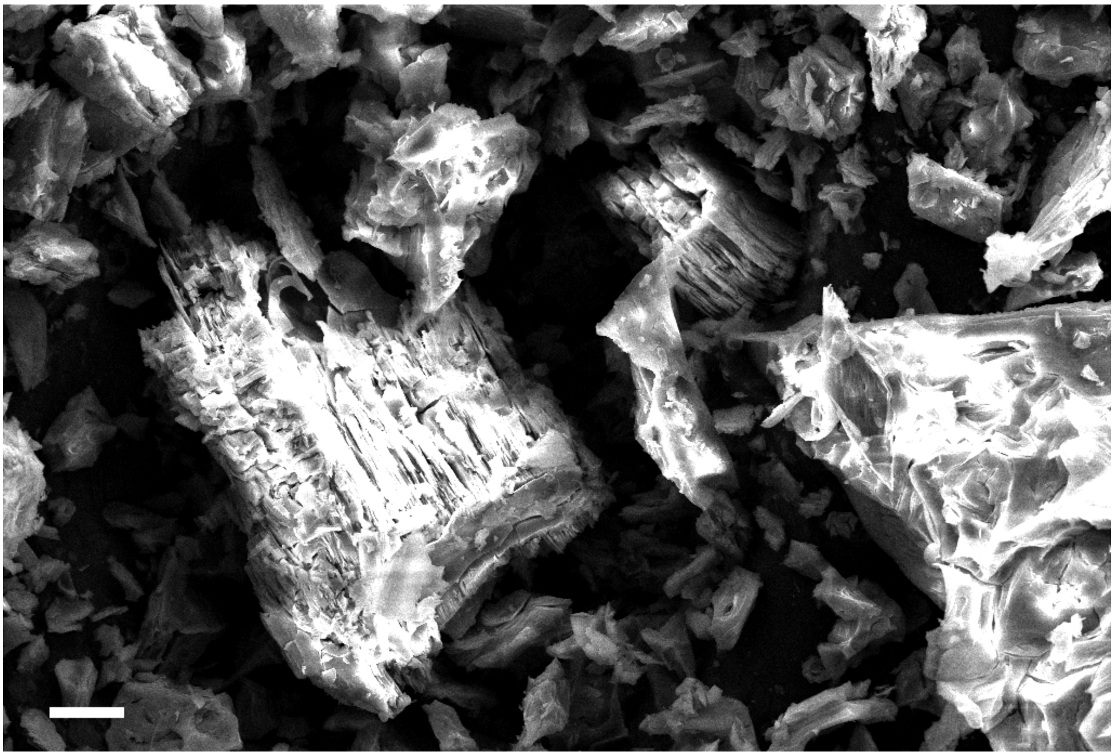

Supplementary Figure 13 | SEM image of the Al_2O_3. Al_2O_3 obtained by heating the $AlCl_3 \cdot 6H_2O$ crystals in a glass bottle for approximately five minutes using an alcohol lamp. Scale bar, 20 μm.

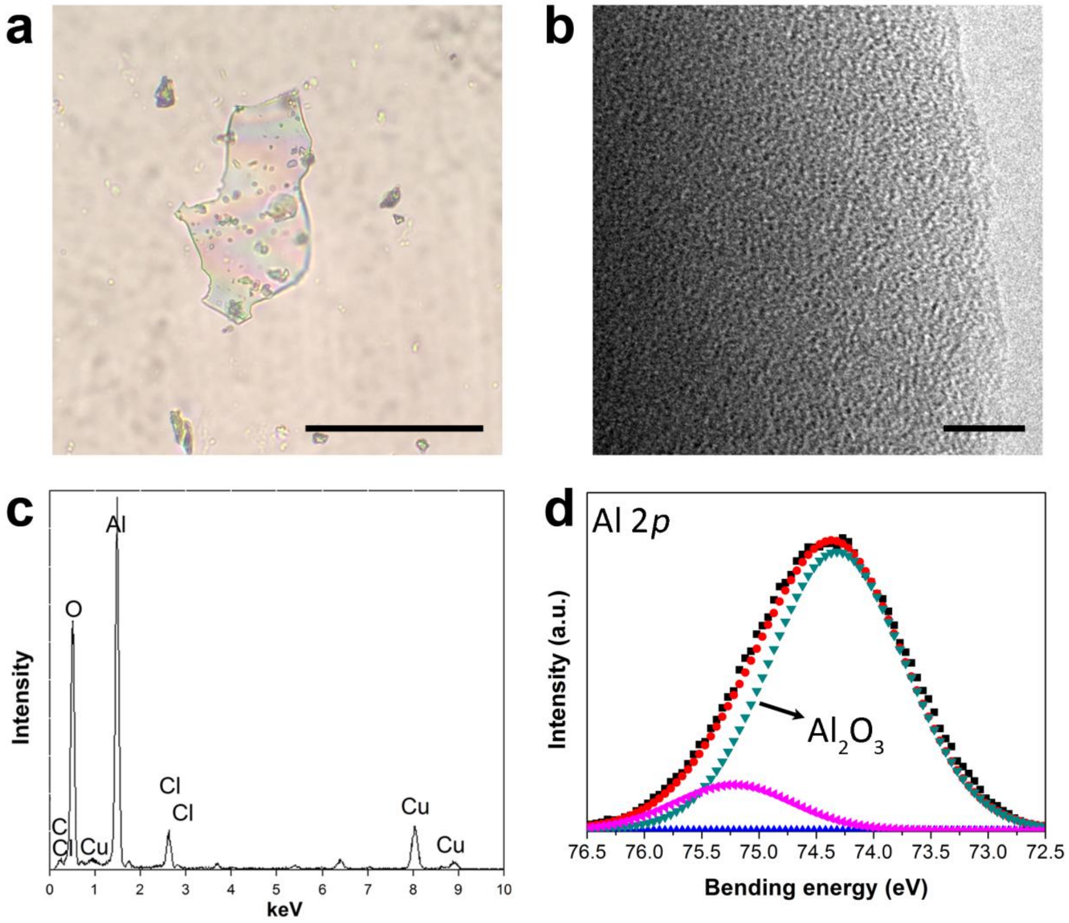

Supplementary Figure 14 | Characterization of Al_2O_3. Al_2O_3 was obtained by heating $AlCl_3 \cdot 6H_2O$ using a heating gun**.** (**a**) Optical microscopy images of Al_2O_3. (**b**) HRTEM image of Al_2O_3 nanosheets. (**c**) EDS result for the Al_2O_3. (**d**) XPS result of the Al_2O_3. Scale bar, 50 μm (**a**) and 10 nm (**b**).

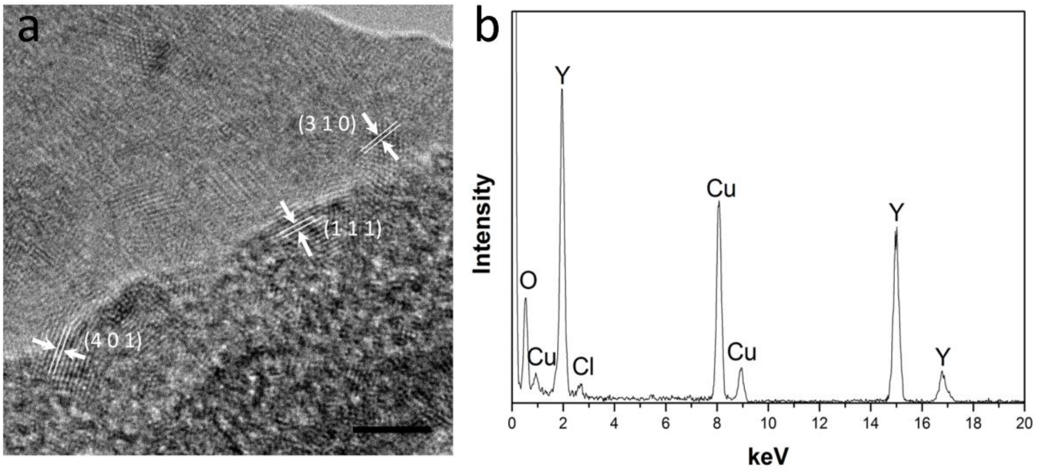

Supplementary Figure 15 | Characterization of Y_2O_3. (**a**) HRTEM image of Y_2O_3 nanosheets. (**b**) EDS result for the Y_2O_3. Scale bar, 5 nm (**a**).

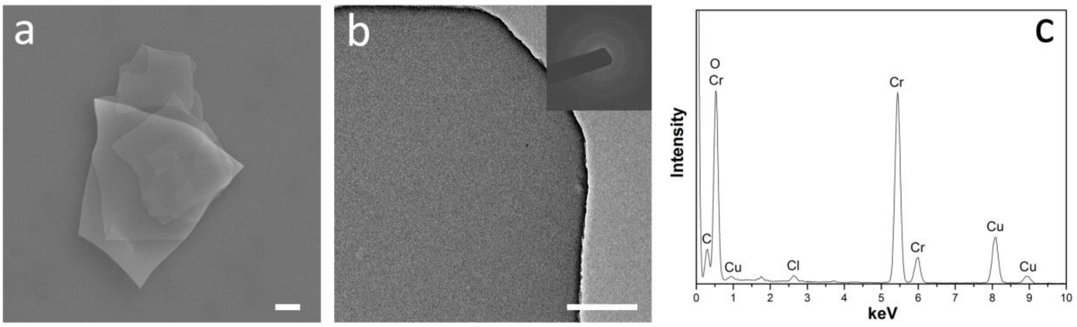

Supplementary Figure 16 | Characterization of the Cr_2O_3. Cr_2O_3 was obtained using a rapid thermal oven. (**a**) SEM (**b**) TEM (the inset is corresponding electron diffraction pattern) and (**c**) EDS results. Scale bar, 2 μm (**a**) and 200 nm (**b**).

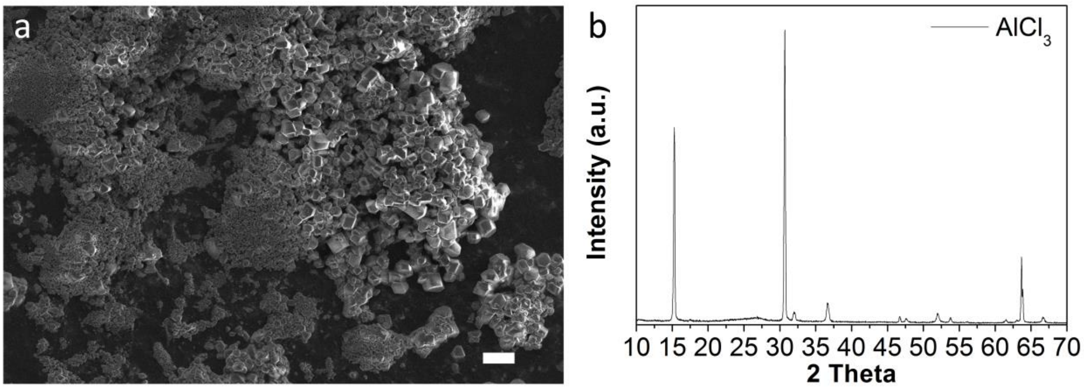

Supplementary Figure 17 | The products fabricated from anhydrous $AlCl_3$. (**a**) SEM image and (**b**) XRD pattern of the products fabricated from anhydrous $AlCl_3$ by rapid heating process (heated the anhydrous $AlCl_3$ using the alcohol lamp). Scale bar, 10 μm. During the heating process, the $AlCl_3$ was volatilized without chemical reaction. Accordingly, XRD unmodified anhydrous $AlCl_3$ after treatment. The obtained product was composed of micron-sized particles without 2D structure.

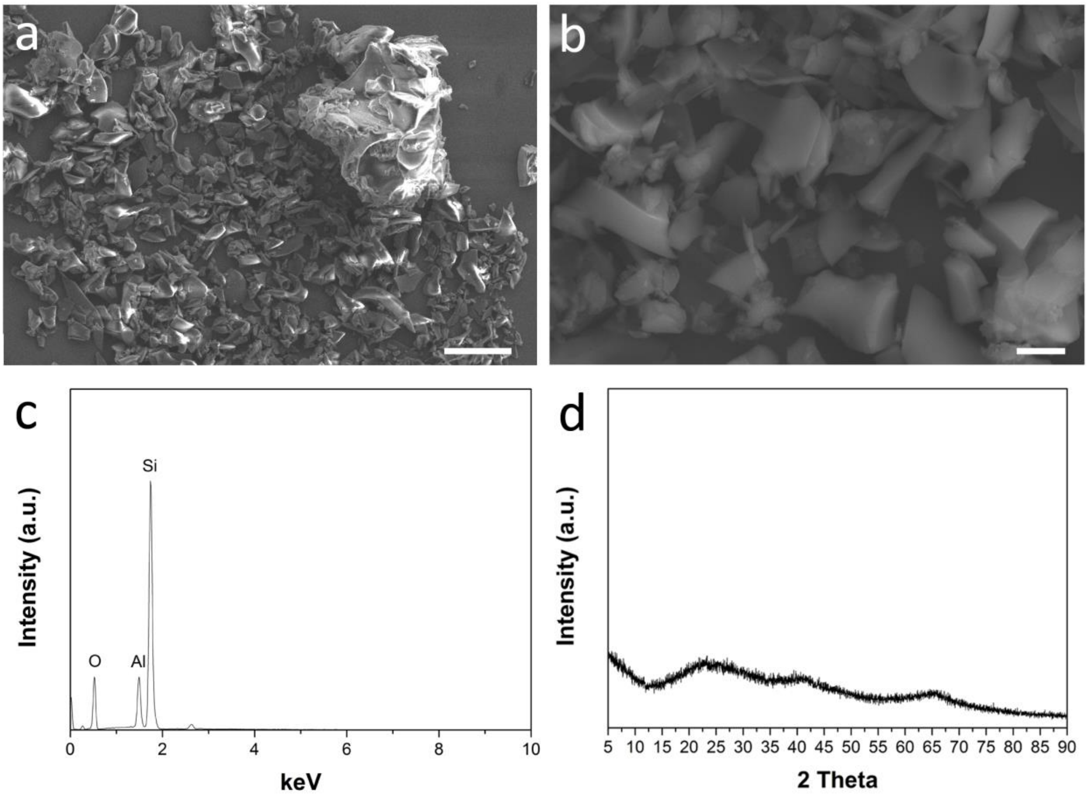

Supplementary Figure 18 | The products fabricated from $AlNO_3$·$9H_2O$. (**a,b**) SEM images, (**c**) EDS result and (**d**) XRD pattern of the products fabricated from $AlNO_3$·$9H_2O$ by rapid heating (heated for approximately five minutes using alcohol lamp). According to the XRD, EDS and SEM results, the obtained amorphous aluminum oxide was micron-sized particles without obvious 2D structure. Scale bar, 10 μm (**a**) and 2 μm (**b**).
